# Peripheral blood TCR repertoire improves early detection across multiple cancer types utilizing a cancer predictor

**DOI:** 10.3389/fonc.2025.1625369

**Published:** 2025-08-27

**Authors:** Yinglei Tang, Xinyi Liao, Bo Liao, Dejun Peng, Qingbo Li

**Affiliations:** ^1^ School of Mathematics and Statistics, Hainan Normal University, Haikou, China; ^2^ China Unicom (Hainan) Industrial Internet Co. Ltd, Haikou, China; ^3^ Key Laboratory of Data Science and Intelligence Education, Hainan Normal University, Ministry of Education, Haikou, China

**Keywords:** TCR repertoire, peripheral blood, cancer detection, deep learning, TCR

## Abstract

**Introduction:**

In the early asymptomatic stages of cancer, the immune system initiates a targeted response against tumor-associated antigens. During this phase, the immune system specifically identifies tumor antigens and triggers the clonal expansion of tumor antigen-specific T cells, which recognize tumor antigen peptides presented by the major histocompatibility complex via the T-cell receptor (TCR) on their surface. Consequently, monitoring alterations in the TCR repertoire holds promise for evaluating an individual’s immune status for cancer detection.

**Methods:**

In this study, we introduced a deep learning framework named DeepCaTCR, designed to enhance the prediction of cancer-associated T-cell receptors. The framework employs a one-dimensional convolutional neural network with variable convolutional kernels, a bidirectional long short-term memory network, and a self-attention mechanism to facilitate feature extraction from amino acid fragments of varying lengths.

**Results:**

DeepCaTCR demonstrates superior performance in cancer-associated TCR recognition, achieving an area under the receiver operating characteristic curve (AUC) of 0.863 and an F1-score of 0.669, thereby outperforming prevailing deep learning models. Validation result indicates that DeepCaTCR effectively distinguishes between tumor-infiltrating lymphocytes (TILs) and healthy peripheral blood samples, achieving an AUC greater than 0.95. It also exhibits high sensitivity (62.5%) and specificity (over 98%) in peripheral blood testing for early-stage cancer patients. To further enhance detection efficacy, we introduced a variance-based repertoire scoring strategy to quantify the dynamic heterogeneity of TCR clonal amplification, resulting in an increased AUC of 0.967 for pan-cancer early screening.

**Discussion:**

This study introduces a novel tool for analyzing the tumor immune microenvironment, offering significant translational potential for early cancer diagnosis. Its key feature is a new scoring method based on variance, not the average method.

## 1 Introduction

The high mortality rate of cancer is primarily due to the late-stage diagnosis of many cases, which consequently leads to lost opportunities for early intervention and treatment. Early cancer screening is as crucial for decreasing both the incidence and mortality rates associated with cancer (1, 2). Traditional imaging methods like endoscopy, CT (3), MRI, and PET (4) are limited to detecting visible cancerous lesions and face challenges in speed, sensitivity, and effectiveness (5). Similarly, tumor marker screenings, such as carcinoembryonic and carbohydrate antigen tests (6), are practical but lack specificity due to the absence of unique markers for many cancer types. Advancements in Artificial Intelligence (AI) have enhanced early cancer screening by creating diagnostic models using tumor marker concentrations (7, 8). Circulating free DNA is a key tool in cancer detection (9), but its plasma concentration can be obscured by noise, complicating early cancer detection. Additionally, the immune system’s response to early-stage cancers produces immune characteristics that, when combined with AI, could serve as immune biomarkers for intelligent early screening models (10, 11).

The tumor microenvironment (TME) is vital in influencing the immune response to cancer by modulating T-cell activity (12). Antigen-specific T cells in the TME are crucial for identifying and attacking tumor antigens (13), aided by the diverse and adaptable T-cell receptor (TCR) repertoire. This diversity is key for effectively targeting cancer cells (14). The expansion and diversification of the TCR repertoire enable T cells to recognize tumor antigens and activate them. Analyzing the TCR repertoire is a powerful approach to understanding the clonal responses of tumor-reactive T cells (15), which are crucial for effective antitumor immune responses. The TCR repertoire provides a detailed map of the diversity and specificity of T cells, which can be used to track the dynamics of immune responses in cancer. Recent advancements in sequencing technologies have enabled the comprehensive analysis of TCR repertoires (16), allowing researchers to identify specific T-cell clones that are reactive to tumor antigens and to understand their role in the immune response against cancer (17). A study demonstrated that the oligoclonal expansion of TCR β clonotypes is associated with effective immune checkpoint therapy responses, suggesting that specific TCR signatures can serve as biomarkers for predicting treatment outcomes (18).

Numerous computational approaches have been devised to detect cancer-associated sequences and estimate cancer probability. However, the identification of cancer-associated T-cell receptors (caTCRs) through computational methods encounters three primary challenges: 1) the presence of weak immune signals attributable to the low neoantigen burden characteristic of early-stage tumors, 2) the conservation of TCR motifs across various cancer types, and 3) the sparse distribution of informative TCR sequences. Although current methodologies offer partial solutions to these challenges, they continue to exhibit significant limitations. Beshnova et al. used convolutional neural networks to differentiate cancer TCRs but covered limited data (19). Xu et al. (20) and Qian et al. (21) employed an enhanced TextCNN network with 1-max pooling and manual filter allocation to identify breast cancer and lung cancer, which may result in the loss of key long-range motifs. Zhang et al. used a pre-trained protein language model to capture TCR sequence features, but its early cancer detection sensitivity is limited by training data bias (22). Cai et al. showed good performance in pan-cancer screening but struggled with early immune microenvironment features (23).

To overcome these challenges, we proposed DeepCaTCR, a deep learning framework that integrates three key innovations. First, we employed multi-scale k-max pooling to capture variable-length motifs (two to five amino acids) while preserving the top k informative segments per filter. Unlike 1-max pooling (in DeepLION, DeepLION2, and BertTCR), this approach mitigates bias toward dominant but non-specific signals and enhances sensitivity to sparse caTCR features. Second, we introduced context-aware feature fusion via bidirectional long short-term memory (LSTM) (BiLSTM) layers, modeling dependencies between discontinuous TCR segments to address motif conservation variability. Third, we implemented a noise-resistant attention mechanism [multi-head self-attention (MHSA)] after k-max pooling to dynamically weight informative sequence regions, suppressing noise from non-cancerous motifs. Our approach uniquely combines these components to enhance caTCR detection in early-stage tumors.

## 2 Materials and methods

In this study, we developed the deep learning framework DeepCaTCR, which effectively manages the varying lengths of amino acid fragments in TCR sequences. Initially, we *de novo* assembled cancer-associated TCRs from RNA-seq data and collected non-cancer TCRs from healthy individuals to create a training dataset. Subsequently, we constructed a pattern recognition network utilizing deep learning algorithms to extract features from amino acid fragments of differing lengths. Finally, we implemented a variance repertoire scoring strategy to quantify individual cancer scores. This study differentiates between cancerous and healthy individuals based on TCR repertoire derived from TCR-seq, exploring non-invasive early cancer detection methods.

### 2.1 Datasets

#### 2.1.1 TCR training data and data processing

The positive training data were generated from CDR3s identified by TRUST (24) from The Cancer Genome Atlas (TCGA) 4,200 tumor RNA-seq samples across 32 cancer types (25). Detailed information on the specific samples is available in **Supplementary Table 1**. This approach was chosen instead of utilizing TCR repertoires from tumor or blood cancer sources. These *de novo* assembled caTCRs from RNA-seq data showed higher specificity than those from TCR-seq data. Only the TCR β chain CDR3 region, crucial for antigenic specificity, was used. TRUST-assembled CDR3 sequences excluded incomplete sequences (not starting with C and ending with F), non-productive sequences (containing B and *), those common in healthy individuals, and sequences shorter than 10 or longer than 24. Negative data from the training set were derived from TCR-seq data of healthy individuals’ peripheral blood (26) by selecting CDR3 sequences with clonal frequencies at least four times the minimum in each TCR repertoire and clustering them using iSMART (27). Incomplete, unproductive, and improperly sized sequences were excluded. This process yielded 30,000 cancer-associated and 59,851 normal CDR3 sequences, mostly ranging from 11 to 20 in length. In this study, only sequences of length 11 to 20 were used for training and validation.

#### 2.1.2 TCR repertoire data and data processing

The TCR cohort repertoire data utilized in this study were obtained from bulk TCR sequencing. The cancer tumor-infiltrating lymphocyte (TIL) cohort comprises samples from breast cancer (BRCA) (28), lung metastasis (Lung BM) (29), lung cancer (29), melanoma (MELA) (30), and pancreatic cancer (PC) (31). The cancer peripheral blood mononuclear cell (PBMC) cohort includes samples from BRCA (28), MELA (32), ovarian cancer (OV) (33), PC (31), colorectal cancer (CRC) (34), bladder cancer (35), glioblastoma multiforme (GBM) (36), and lung cancer (37). The cancer staging PBMC cohort encompasses stage I–II lung cancer (38), stage III lung cancer (38), stage I renal cell carcinoma (RCC) (19), borderline ovarian cancer (19), stage II–III ovarian cancer (19), and stage II PC (19). The non-cancer PBMC cohorts consist of samples from yellow fever virus (YFV) (39), human cytomegalovirus (HCMV) (26), healthy T-cell controls (Healthy TC) (40), graft-versus-host disease (GVHD) (41), healthy donors (HCMV−) (26), and healthy donors (42). The details of the datasets are provided in **Supplementary Table 1**. In the preprocessing of repertoire data, TCR sequences with lengths ranging from 11 to 20 nucleotides were selected. Following the exclusion of unqualified TCR sequences as detailed in Section 3.1, the sequences with the top 10,000 clone scores were retained for further analysis. These sequences were subsequently clustered using the iSMART algorithm. The TCR sequences resulting from this clustering process were considered in this study to be those most likely associated with cancer.

### 2.2 Multi-scale attentive BiLSTM for TCR motif analysis

**Figure 1:**
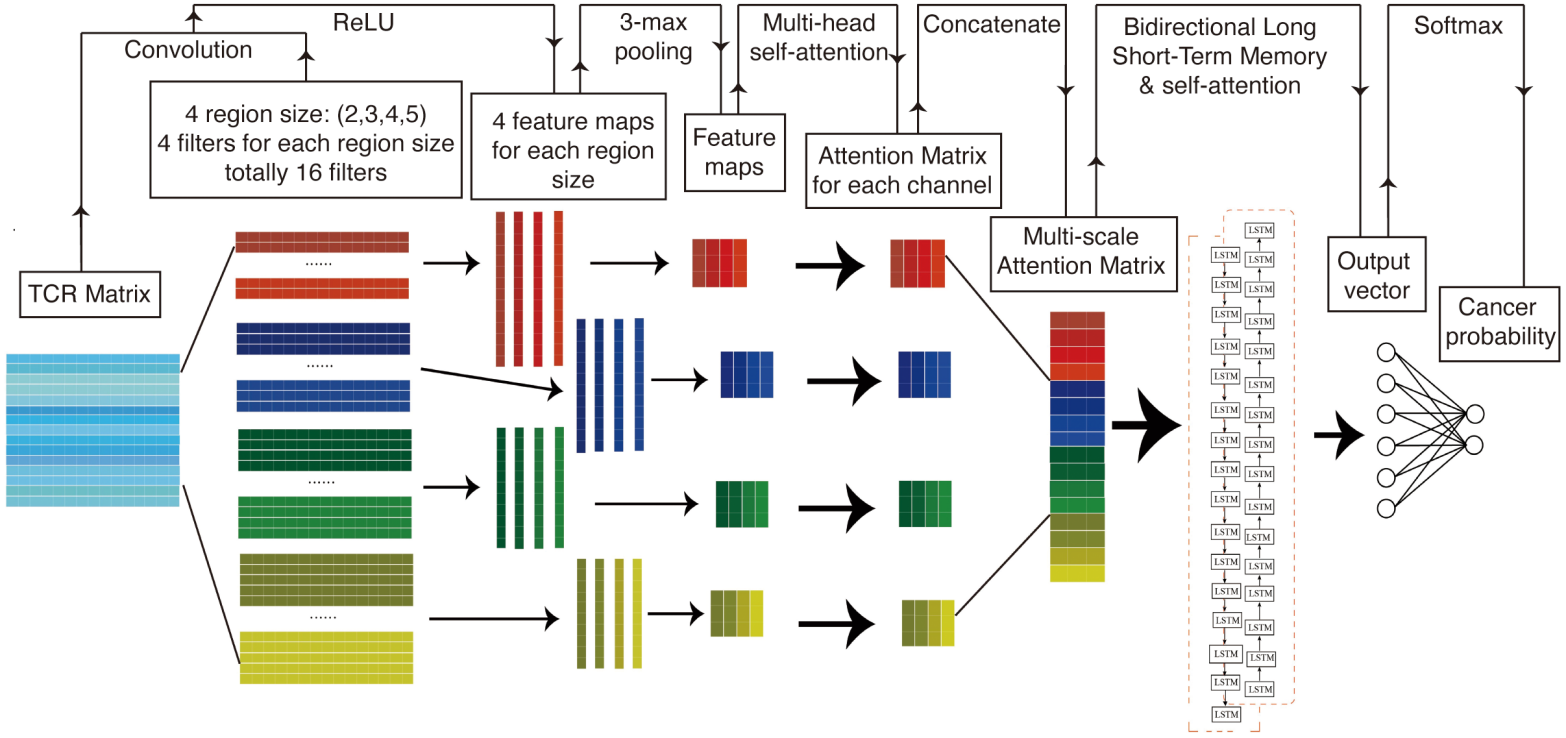
Structure diagram of TCR sequence recognition algorithm. TCR, T-cell receptor.

**Figure 1** presents a structural diagram of the TCR sequence recognition algorithm. In summary, TCR sequences associated with cancer and those not associated with cancer are initially encoded into a matrix using amino acid biochemical features as model inputs. This matrix is subsequently processed in the convolutional layer using a multi-scale convolutional kernel to extract features. A max pooling layer is employed to encode the feature set of amino acid fragments of varying lengths before applying a multi-head self-attention mechanism to assign differential attentional weights. The resulting attention-weighted encoding matrices are interconnected along the channel dimension, producing an attention-weighted matrix that contains key molecules of different lengths. This weighted pattern matrix is then further processed using bidirectional long- and short-term memory networks, which focus on the correlations between these key patterns. Finally, a self-attention mechanism is introduced to assign varying attention weights, followed by the application of a linear classifier for binary classification.

#### 2.2.1 1D convolutional neural network

Deep convolutional neural networks (CNNs) are a class of deep learning algorithms adept at identifying latent patterns within grid data. CNNs serve as highly effective tools for feature extraction from such data, often outperforming traditional machine learning algorithms (23). However, when CNNs are employed to extract features from equal-length sequence encoding matrices, created by padding variable-length sequences with zero vectors, the model performance tends to degrade. This degradation is likely due to the introduction of zero vectors via AA index encoding, which alters the original data length distribution and introduces significant noise. To mitigate this issue, we used a one-dimensional CNN (1D CNN) algorithm to transform the encoding matrix into a one-dimensional sequence. This approach more effectively preserves sequence information and the dependencies between sequences (19, 21). Let the input sequence be represented as a matrix $X\in {\mathbb{R}}^{L\times d}$ , where *L* is the padded sequence length and *d* is the encoding dimension (amino acid index features). The 1D convolution operation applies a filter $W_{C}\in {\mathbb{R}}^{k\times d}$ with kernel size *k*, sliding over the sequence to generate feature maps (Equation 1):


(1) $$h_{C}^{\left(i\right)}=ReLU\left(W_{C}\cdot X_{i:i+k-1}+b_{C}\right)$$

where $X_{i:i+k-1}$ is the subsequence window from position *i* to *i + k* − 1, and $b_{C}$ is a bias term.

#### 2.2.2 k-max pooling

Furthermore, we employed the *k*-max pooling algorithm to transform the one-dimensional coding sequence into a sequence of uniform length, effectively mitigating interference from zero vector padding, and *k*-max pooling selects the k largest values from the feature map $h_{C}$ (Equation 2):


(2) $$P_{k}=Top_{k}\left(h_{C}\right)$$

where $Top_{k}$ retains the *k* highest activations.

#### 2.2.3 Multi-scale convolutional kernels

The currently employed algorithm is limited to acquiring amino acid fragments of a fixed length from the sequence. However, prior research has demonstrated that the length of cancer-related key motifs is variable, typically ranging from two to eight amino acids. To capture the characteristics of amino acid fragments of varying lengths, this study adapted the TextCNN model from natural language processing, implementing convolutional kernels of diverse sizes within the convolutional layer (20). To capture motifs of variable lengths (*k*_1_, *k*_2_, …, *k*_n_), parallel convolutional kernels of different sizes are applied (Equation 3):


(3) $$H_{multi}={⊕}_{j=1}^{n}H_{kj}$$

where $H_{kj}$ is the feature map from the *j*th kernel and ⨁ denotes concatenation along the channel dimension.

#### 2.2.4 Self-attention mechanism

In the context of the weighted motif matrix of a sequence, it is acknowledged that amino acid fragments of varying lengths exert differential influences on sequence specificity. To address this, a self-attention mechanism was implemented to evaluate the similarity between different positions within the sequence, assigning an attention weight to each position. This allows the model to autonomously identify the key motifs within the sequence. Given the multi-scale feature matrix $H_{multi}$ , the attention weights $\alpha_{i}$ for each position *i* are computed as follows (Equation 4):


(4) $$\alpha_{i}=softmax(\frac{QK^{T}}{\sqrt{d_{k}}})$$

where $Q=H_{multi}W_{Q}$ , $K=H_{multi}W_{K}$ , and $W_{Q}$ , $W_{K}$ are learnable query/key matrices. The attention-weighted output is as follows (Equation 5):


(5) $$A=\sum_{i=1}^{L}\alpha_{i}H_{multi}^{\left(i\right)}$$

#### 2.2.5 Bidirectional long short-term memory

Nonetheless, it has been observed that this algorithmic approach neglects the interconnections between key motifs within the same sequence. LSTM networks, a class of neural networks specifically designed for sequential data processing, offer a potential solution. In LSTM networks, the output at each time step, known as the hidden state, encapsulates all input information up to that point. Additionally, the cell state serves as a repository for long-term information. The input gate computes an activation value based on the current input and the state from the preceding moment to determine the acceptance of new input. Similarly, the forgetting gate calculates the degree of forgetting by evaluating the current input alongside the previous state. Activation values for each gate are computed based on the hidden state from the preceding moment.

In contrast, the BiLSTM model processes sequential data by considering not only the current position at each time step but also both preceding positions (via the forward LSTM) and subsequent positions (via the backward LSTM). This dual processing results in the generation of two hidden states at each time step: one derived from the forward network and the other from the backward network. These hidden states are subsequently combined to form a comprehensive context representation that encapsulates enduring dependency information within the text. Consequently, this model is capable of capturing more profound contextual associations. BiLSTM processes the attention-weighted matrix $A\in {\mathbb{R}}^{L\times m}$ to model long-range dependencies (44). For each time step *t*, the forward ( ${\vec{h}}_{t}$ ) and backward ( ${\overset{\leftarrow }{h}}_{t}$ ) hidden states are computed as follows (Equation 6):


(6) $${\vec{h}}_{t}=LSTM\left(A_{t},{\vec{h}}_{t-1}\right),{\overset{\leftarrow }{h}}_{t}=LSTM\left(A_{t},{\overset{\leftarrow }{h}}_{t+1}\right)$$

The final hidden state combines both directions (Equation 7):


(7) $$h_{t}=\left[{\vec{h}}_{t}∥{\overset{\leftarrow }{h}}_{t}\right]$$

where || denotes concatenation.

#### 2.2.6 Classification layer

Prior to the introduction of the attention mechanism, we input the weighted motif matrix into BiLSTM to evaluate the correlation between different key motifs of the sequence, thereby adaptively capturing the long-range dependencies between amino acid fragments. The aggregated hidden states $H_{BiLSTM}\in {\mathbb{R}}^{L\times 2m}$ are fed into a fully connected layer with softmax for binary classification (Equation 8):


(8) $$\hat{y}=softmax\left(W_{f}\cdot Flatten\left(H_{BiLSTM}\right)+b_{f}\right)$$

where $W_{f}$ and $b_{f}$ are learnable parameters.

### 2.3 Cancer predictor

#### 2.3.1 TCR repertoire mean scoring strategy

Let *R* = {TCR_1_, TCR_2_, …, TCR*_N_*} represent a TCR repertoire containing *N* distinct TCRs, and the composite score of the TCR repertoire $S\left(R\right)$ is defined as the arithmetic mean of the predicted cancer scores across all TCRs in *R* (Equation 9):


(9) $$S\left(R\right)=\frac{1}{N}\sum_{i=1}^{N}f\left(TCR_{i}\right)$$

where $f\left(TCR_{i}\right)$ denotes the predicted cancer score of the *i*th TCR (*i* = 1, 2, …, *N*). This formulation reflects the intuition that the overall repertoire score represents the average likelihood of cancer-associated specificity across its constituent TCRs.

#### 2.3.2 TCR repertoire variance scoring strategy

Let *R* = {TCR_1_, TCR_2_, …, TCR*_N_*} represent a TCR repertoire containing *N* distinct TCRs, and the variance-based composite score $V\left(R\right)$ is then defined as the variance of the predicted cancer scores across all TCRs in *R* (Equation 10):


(10) $$V\left(R\right)=\frac{1}{N}\sum_{i=1}^{N}{\left(f\left(TCR_{i}\right)-\mu \left(R\right)\right)}^{2}$$

where $\mu \left(R\right)$ is the mean predicted cancer score (as defined in the mean strategy). This formulation quantifies the spread (heterogeneity) of predicted cancer scores within the repertoire, with higher variance indicating greater diversity in cancer-associated specificity among TCRs.

### 2.4 TCR sequence recognition model parameter settings

The model architecture and final hyperparameter configuration, including convolutional kernel dimensions, pooling strategies, and fully connected layer specifications, are detailed in **Table 1**. The process of parameter tuning, which involves a systematic evaluation of alternative dropout rates and learning rates, along with the associated performance metrics, is thoroughly documented in **Supplementary Table 2**.

**Table 1:** TCR sequence recognition model architecture and hyperparameters.

Layer/component	Parameter setting
Input encoding	1. TCR sequence encoded as L × 15 matrix. 2. Zero-padded to 20 × 15 if L < 20.
Multi-scale convolution	1. Kernel widths: fixed at 15 (matches input dimension). 2. Kernel heights: 2, 3, 4, 5. 3. Kernels per height: 4 (total 16 kernels).
Max pooling	1. Window size: 3. 2. Output: 3 × 4 matrix *P*.
Multi-head self-attention	1. Attention heads: 2. 2. Hidden dimension: 4 (aligned with P). 3. Subspace projection for *Q*, *K*, *V*.
Bidirectional LSTM	1. Input dimension: 3. 2. Hidden dimension: context-aware (self-attention adjusted). 3. Output: concatenated forward/backward states.
Fully connected layer	1. Units: 6. 2. Dropout: 50% regularization. 3. Activation: softmax (binary classification).
Output	Probabilities for cancer/non-cancer classes.

LSTM, long short-term memory.

### 2.5 Model training and evaluation

The experiments were executed on a high-performance computing platform operating Ubuntu 20.04, featuring an Intel^®^ Xeon^®^ Platinum 8470Q processor with 20 virtual CPUs, 90GB of RAM, and an NVIDIA virtual GPU with 48GB of memory. The software environment consisted of Python 3.8 and PyTorch 1.10.0 with CUDA 11.3 for acceleration, supplemented by standard scientific computing libraries. For model development, 30,000 cancer-associated CDR3 sequences and approximately 60,000 non-cancer sequences were encoded, assigning binary labels (1 for cancer and 0 for non-cancer). The dataset was divided using stratified sampling, with 80% designated for training and 20% for validation. To ensure robust performance evaluation, fivefold cross-validation was employed across all experiments. The training process utilized the Adam optimizer with a learning rate of 0.001 and cross-entropy loss for error computation. To mitigate overfitting, dropout was applied with a probability of 0.5 during training. The model was trained for a maximum of 1,000 epochs, with an early stopping criterion activated if the validation loss did not improve for 20 consecutive epochs.

### 2.6 Validation metrics

This study utilized six metrics to assess model performance: accuracy (ACC), sensitivity (SEN), specificity (SPE), area under the receiver operating characteristic curve (AUC), F1-score, and Matthews Correlation Coefficient (MCC). Each metric offers unique insights into the classifier’s capabilities (Equations 11–17):


(11) $$ACC=\frac{TP+TN}{TP+TN+FP+FN}$$

(12) $$SEN=\frac{TP}{TP+FN}$$

(13) $$SPE=\frac{TN}{TN+FP}$$

(14) $$precision=\frac{TP}{TP+FP}$$

(15) $$Recall=\frac{TP}{TP+FN}$$

(16) $$F1_{score}=\frac{2\times precision\times recall}{precision+recall}$$

(17) $$MCC=\frac{TP\times TN-FP\times FN}{\sqrt{\left(TP+FP\right)\left(TP+FN\right)\left(TN+FP\right)\left(TN+FN\right)}}$$

where *TP*, *TN*, *FP*, and *FN* represent true-positive, true-negative, false-positive, and false-negative predictions, respectively.

## 3 Results

### 3.1 Model performance in recognizing caTCRs

Due to the inability to directly utilize raw amino acid sequences for model training, this study employed a biochemical feature-based encoding strategy to convert these sequences into numerical form. Focusing on the functional characteristics of antigen-binding sites within the CDR3 region, 553 biochemical feature indicators of amino acids were selected from the AAindex database for principal component analysis (PCA). Through dimensionality reduction, a 20 × 20 amino acid feature matrix was derived, and the top 15 principal components, which collectively accounted for over 95% of the cumulative variance, were chosen to construct a standardized 20 × 15 AAindex coding matrix. For CDR3 sequences shorter than 20 amino acids, a zero-padding strategy was applied to encode them uniformly into a 20 × 15 matrix structure.

In order to assess the performance of DeepCaTCR, we conducted a comparative analysis with leading caTCR recognition models, namely, DeepLION and BertTCR. This evaluation utilized a consistent encoding scheme, training dataset, learning rate, and batch size across all models. Through fivefold cross-validation, DeepCaTCR demonstrated superior performance in antigen-specific TCR recognition, achieving an ACC of 0.807 ± 0.003 and AUC of 0.863 ± 0.003, as presented in **Table 2**. Notably, DeepCaTCR outperformed both DeepLION (ACC: 0.801, AUC: 0.854) and BertTCR (ACC: 0.760, AUC: 0.790), achieving the highest ACC and AUC values. The sensitivity of DeepCaTCR (0.586) was 28% higher than that of BertTCR (0.457), while its specificity (0.918) remained the highest among all models evaluated. Furthermore, the F1-score (0.669) and MCC (0.548) exceeded those of the competing models.

**Table 2:** Performance comparison of caTCR recognition models.

Model	ACC	AUC	SEN	SPE	F1	MCC
DeepCaTCR	0.807 ± 0.003	0.863 ± 0.003	0.586 ± 0.026	0.918 ± 0.010	0.669 ± 0.013	0.548 ± 0.009
DeepLION	0.801 ± 0.003	0.854 ± 0.004	0.577 ± 0.014	0.913 ± 0.009	0.659 ± 0.006	0.533 ± 0.007
BertTCR	0.760 ± 0.003	0.790 ± 0.004	0.457 ± 0.022	0.911 ± 0.009	0.559 ± 0.017	0.425 ± 0.009
DeepCaTCR-noBiLSTM	0.795 ± 0.006	0.848 ± 0.005	0.593 ± 0.032	0.895 ± 0.024	0.657 ± 0.007	0.520 ± 0.008
DeepCaTCR-noMHSA	0.800 ± 0.003	0.853 ± 0.005	0.602 ± 0.013	0.898 ± 0.011	0.666 ± 0.003	0.532 ± 0.007
DeepCaTCR-noBiLSTM-noMHSA	0.791 ± 0.005	0.840 ± 0.006	0.560 ± 0.020	0.906 ± 0.014	0.641 ± 0.008	0.509 ± 0.009

ACC, accuracy; AUC, area under the receiver operating characteristic curve; SEN, sensitivity; SPE, specificity; MCC, Matthews Correlation Coefficient.

**Figure 2:**
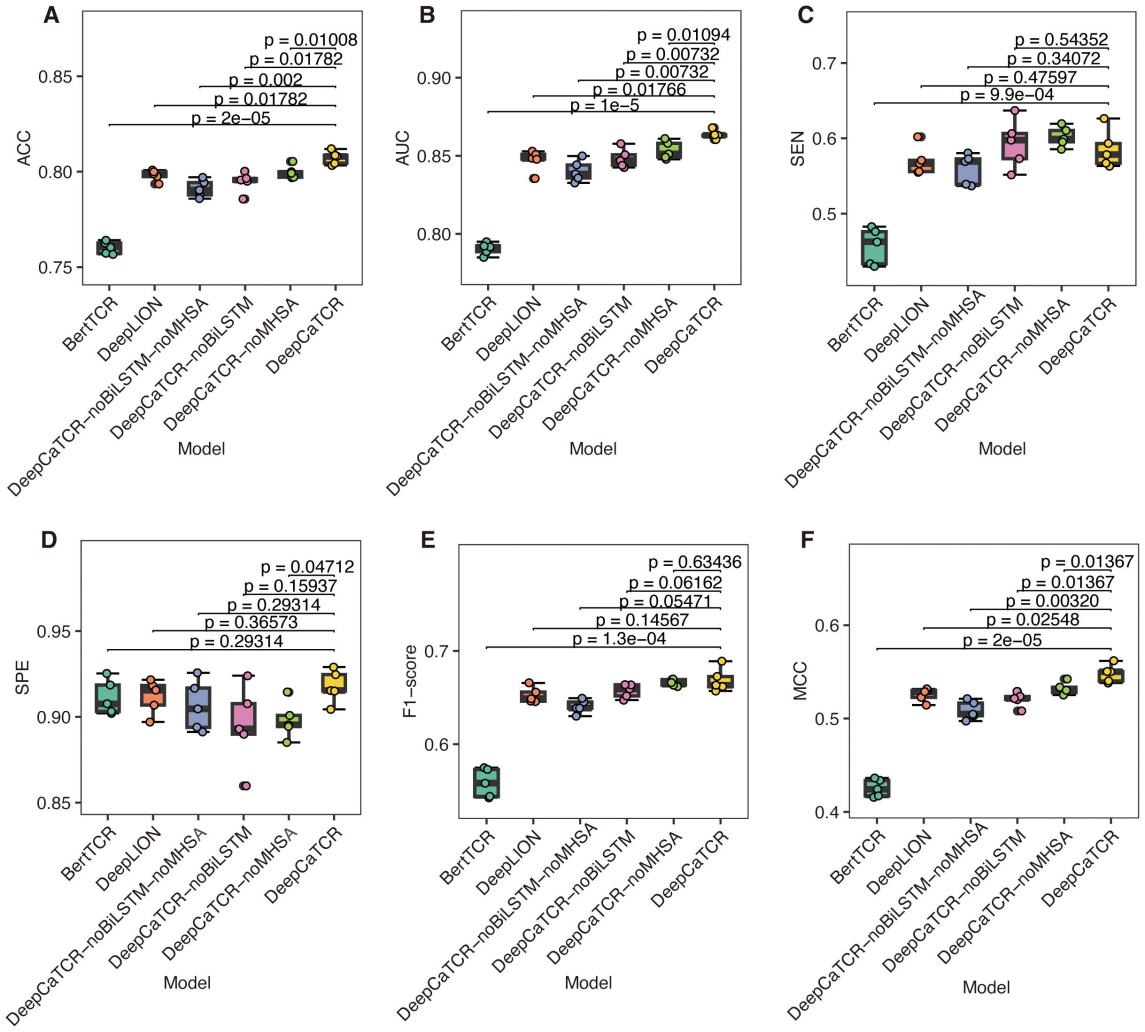
Comparison of model performance using fivefold cross-validation. **(A–F)** The results for six evaluation metrics: **(A)** ACC, **(B)** AUC, **(C)** SEN, **(D)** SPE, **(E)** F1-score, and **(F)** MCC. The models under comparison include DeepCaTCR, DeepCaTCR-noBiLSTM, DeepCaTCR-noMHSA, DeepCaTCR-noBiLSTM-noMHSA, DeepLION, and BertTCR. The box plots depict the distribution of each metric across the five folds, while individual data points indicate the metric value for each fold. Statistical significance was evaluated using paired t-tests, with each model compared against DeepCaTCR as the reference model. TCR, T-cell receptor; ACC, accuracy; AUC, area under the receiver operating characteristic curve; SEN, sensitivity; SPE, specificity; MCC, Matthews Correlation Coefficient.

We conducted a detailed analysis of the performance metrics for each fold and performed paired t-tests to assess statistical significance, comparing each model against DeepCaTCR. The findings indicated that BertTCR was significantly outperformed by DeepCaTCR across all metrics (p < 0.0001), with the exception of specificity (**Figure 2**). DeepLION demonstrated significantly lower performance than DeepCaTCR in terms of ACC, AUC, and MCC (p = 0.01–0.02). We posited that the suboptimal performance of BertTCR could be attributed to its limited number of filters, which was initially set at six. To investigate this hypothesis, we increased the number of filters in BertTCR to nine, resulting in a significant enhancement in model performance (p < 0.008, **Supplementary Figure S1**).

### 3.2 Functional analysis of DeepCaTCR key modules

To further substantiate the contributions of the core components within DeepCaTCR, we performed ablation studies focusing on BiLSTM and MHSA. We developed variant models, namely, DeepCaTCR-noBiLSTM, DeepCaTCR-noMHSA, and DeepCaTCR-noBiLSTM-noMHSA. We subjected these ablation variants to the same experimental conditions as the baseline DeepCaTCR model, encompassing input data preprocessing, shared embedding layer parameters, output layer architecture, loss function, optimizer configuration, and train/test splits. The sole alteration involved the exclusion of specific model components.

The comprehensive DeepCaTCR model demonstrated superior performance across several metrics, achieving the highest accuracy (0.807), AUC (0.863), specificity (0.918), F1-score (0.669), and MCC (0.548). This underscores the synergistic advantages of integrating BiLSTM and MHSA. Upon the exclusion of BiLSTM (DeepCaTCR-noBiLSTM), there were notable declines in performance metrics: ACC decreased by 1.5%, AUC by 1.7%, SPE by 2.5%, F1 by 1.8%, and MCC by 5.1% (**Table 2**). Interestingly, SEN exhibited a slight improvement (0.593 compared to 0.586), which may be attributed to the reduced complexity of the model influencing class-specific predictions. The removal of MHSA (DeepCaTCR-noMHSA) resulted in smaller yet consistent reductions in ACC (0.9%), AUC (1.2%), SPE (2.2%), F1 (0.4%), and MCC (2.9%). Similar to the removal of BiLSTM, SEN improved (0.602 compared to 0.586), suggesting that attention mechanisms may trade off some sensitivity for specificity. The most pronounced degradation in performance metrics (ACC: −2.0%, AUC: −2.7%, F1: −4.2%, MCC: −7.1%) underscores the complementary roles of BiLSTM and MHSA in feature extraction and context modeling. Notably, SEN experienced a sharp decline (0.560 compared to 0.586), indicating that the combined use of BiLSTM and MHSA enhances recall for positive samples.

### 3.3 Model performance in cancer patient identification

While DeepCaTCR exhibits strong capabilities in recognizing cancer-associated sequences, its effectiveness in clinically distinguishing between cancer patients and healthy individuals requires further validation through independent experiments. It is important to note that accurately evaluating the overall immune status presents substantial technical challenges. This difficulty arises because antigen-specific TCRs constitute only a small fraction of an individual’s TCR repertoire, typically less than 0.1%, and there is a considerable background noise (43). To address this issue, the study employed the iSMART antigen-specific clustering technique to extract representative sequences from each database. This approach enabled the quantification of an individual’s tumor immune response by calculating the mean cancer probability, or cancer score, of these characteristic sequences.

**Figure 3:**
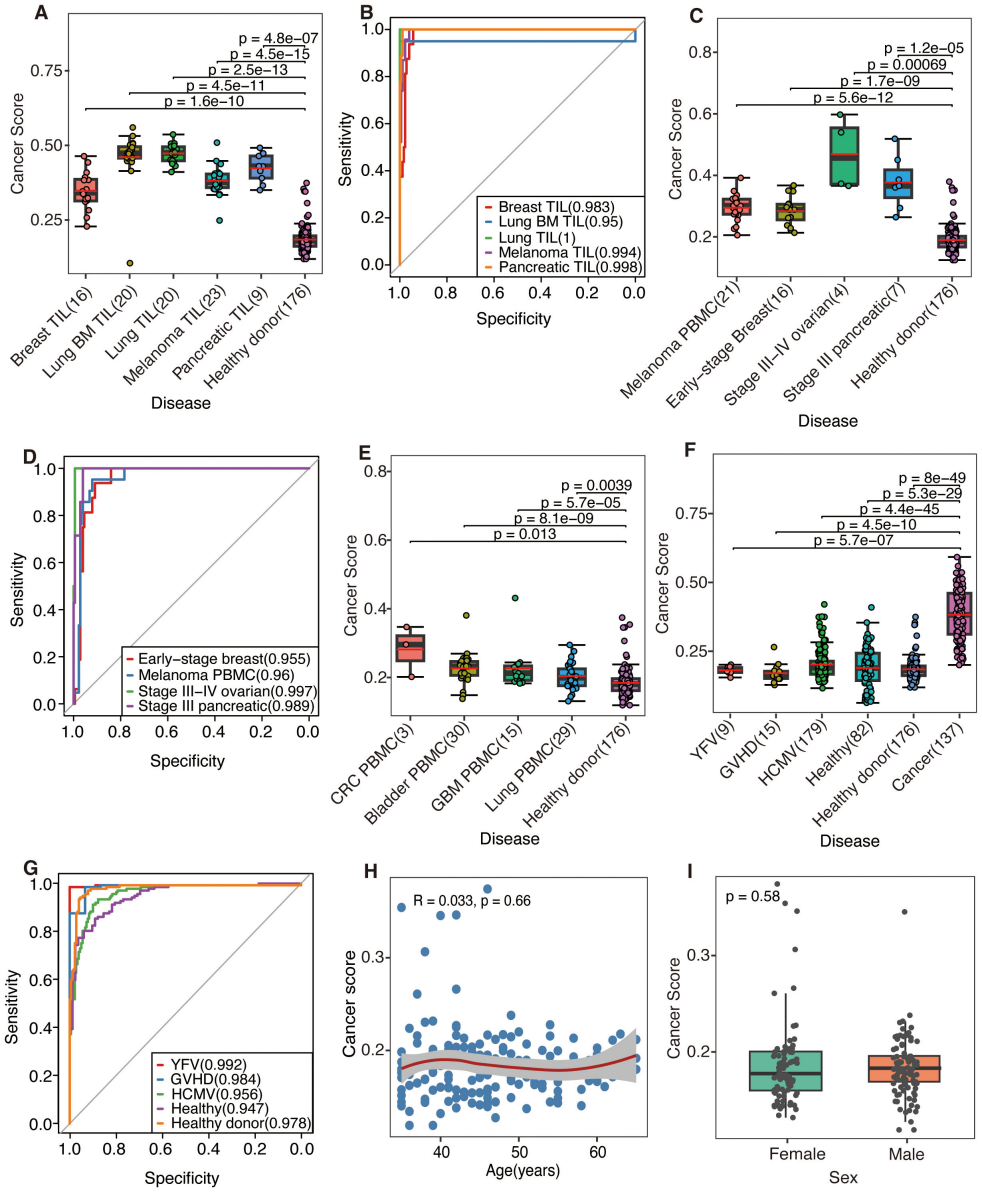
The predictive performance was evaluated by DeepCaTCR utilizing the mean scorer. **(A, C, E)** Box plots and scatter plots illustrating the distribution of cancer scores across various scenarios: **(A)** tumor-infiltrating lymphocytes (TILs) from different cancer types, **(C)** peripheral blood from untreated cancer patients, and **(E)** peripheral blood from treated cancer patients. The sample sizes are indicated on the x-axis. Comparisons were conducted with a cohort of healthy donors (n = 176), and statistical significance was assessed using the Wilcoxon rank-sum test. The red solid line denotes the average predicted score for each donor on the y-axis. **(B, D)** ROC curves and AUC values for cancer patients, using healthy donors (n = 176) as the control group. **(B, D)** The model’s performance in predicting TIL samples and untreated PBMC samples, respectively. **(F)** Box plots and scatter plots that depict the distribution of cancer scores from various virus-infected or healthy donors. Comparisons were made with the untreated cancer cohort (n = 137), employing the same statistical significance assessment method as in panel **(A)**. **(G)** ROC curves and AUC values are presented for distinguishing between different virus-infected and healthy donors, using the untreated cancer cohort (n = 137) as the control group. **(H)** A scatter plot illustrates the association between age (x-axis) and cancer risk score (y-axis) within a cohort of healthy participants (n = 176). LOWESS smooth curve was added on top of the scatter plot to display the trend of change. Spearman’s rank correlation analysis was conducted, with the correlation coefficient (R) and statistical significance presented in the plot inset. **(I)** Comparative analysis of cancer scores between male and female healthy individuals. The p-value derived from the Wilcoxon rank-sum test is indicated on the plot. ROC, receiver operating characteristic; AUC, area under the receiver operating characteristic curve; PBMC, peripheral blood mononuclear cell.

DeepCaTCR demonstrated robust discriminatory power across diverse sample types and clinical scenarios, as quantified by the mean cancer score of antigen-specific TCR clusters. TILs exhibited significantly higher cancer scores than PBMCs from healthy donors (p < 5e−07, Wilcoxon rank-sum test, **Figure 3A**). There was near-perfect discrimination (AUC > 0.95) for all cancer types (**Figure 3B**, **Supplementary Table S3**), with primary lung cancer (AUC = 1), pancreatic cancer (AUC = 0.998), and melanoma (AUC = 0.994) showing high specificity (SPE > 0.96) and sensitivity (SEN = 1.0). Untreated cancer patients had significantly higher PBMC cancer scores than healthy controls (**Figure 3C**, p < 0.0007), with ovarian cancer (AUC = 0.997) and pancreatic cancer (AUC = 0.989) having the leading performance (**Figure 3D**). Treated patients (**Figure 3E**) displayed reduced cancer scores versus untreated cohorts, likely due to the therapy-induced depletion of tumor-reactive T cells. Despite lower scores, the most model-maintained AUC > 0.81 (**Supplementary Table S3**) was for refractory cancers (glioblastoma: AUC = 0.814; bladder cancer: AUC = 0.83; CRC: AUC = 0.919), although lung cancer discrimination declined (AUC = 0.667), potentially reflecting prolonged T-cell exhaustion.

Notably, DeepCaTCR maintained high specificity in non-cancer contexts. Evaluation of virus-infected and healthy cohorts (**Figures 3F, G**, **Supplementary Table 4**) revealed consistently strong performance metrics: YFV (AUC = 0.992, SPE = 1.0), GVHD (AUC = 0.984, SPE = 0.933), and HCMV (AUC = 0.956, SPE = 0.899). Healthy donors (AUC = 0.978, SPE = 0.955) and additional healthy samples (AUC = 0.947, SPE = 0.89) further confirmed the model’s ability to distinguish cancer-associated TCRs from benign immune responses. Preliminary observations suggested that, despite several elevated cancer scores, the scores within the healthy cohort remain relatively low. Subsequent validation using additional cohorts comprising both healthy and virus-infected individuals demonstrated that the cancer scores fall within anticipated ranges (**Figure 3F**), indicating that the initial findings may be attributable to the characteristics of the study population rather than methodological flaws.

Further analysis of the correlation between cancer scores and demographic variables such as age and gender yielded a Spearman’s correlation coefficient of R = 0.033 (p = 0.66, **Figure 3H**), while a comparison by gender using the Wilcoxon test resulted in a p-value of 0.58 (**Figure 3I**), indicating no significant association. Additionally, we examined the correlation between cancer scores and TCR counts. In the initial healthy cohort (n = 176), a marginal correlation was observed (R = 0.15, p = 0.044, **Supplementary Figure S2A**). In the subsequent validation cohort (n = 82), a significant but stronger correlation was found (R = −0.5, p = 2.1e−06, **Supplementary Figure S2B**). However, in the combined analysis (n = 258), no correlation was detected (R = 0.021, p = 0.74, **Supplementary Figure S2C**). The results suggest that cancer scores are generally stable within healthy populations, unaffected by age or gender, and not clearly associated with TCR counts. Elevated scores may reflect population-specific characteristics or weak biological factors.

### 3.4 Model diagnostic performance in early cancer detection

Building on the exceptional recognition performance of peripheral blood samples from early-stage breast cancer patients demonstrated in the previous study (AUC = 0.955), this research further validated the generalizability of DeepCaTCR for the early diagnosis of multiple cancer types. The model’s capability to differentiate between tumor stages was systematically evaluated by collecting PBMC samples from patients with early (stage I–II) and advanced (stage III–IV) primary treatment. Additionally, independent healthy samples were collected as controls.

**Figure 4:**
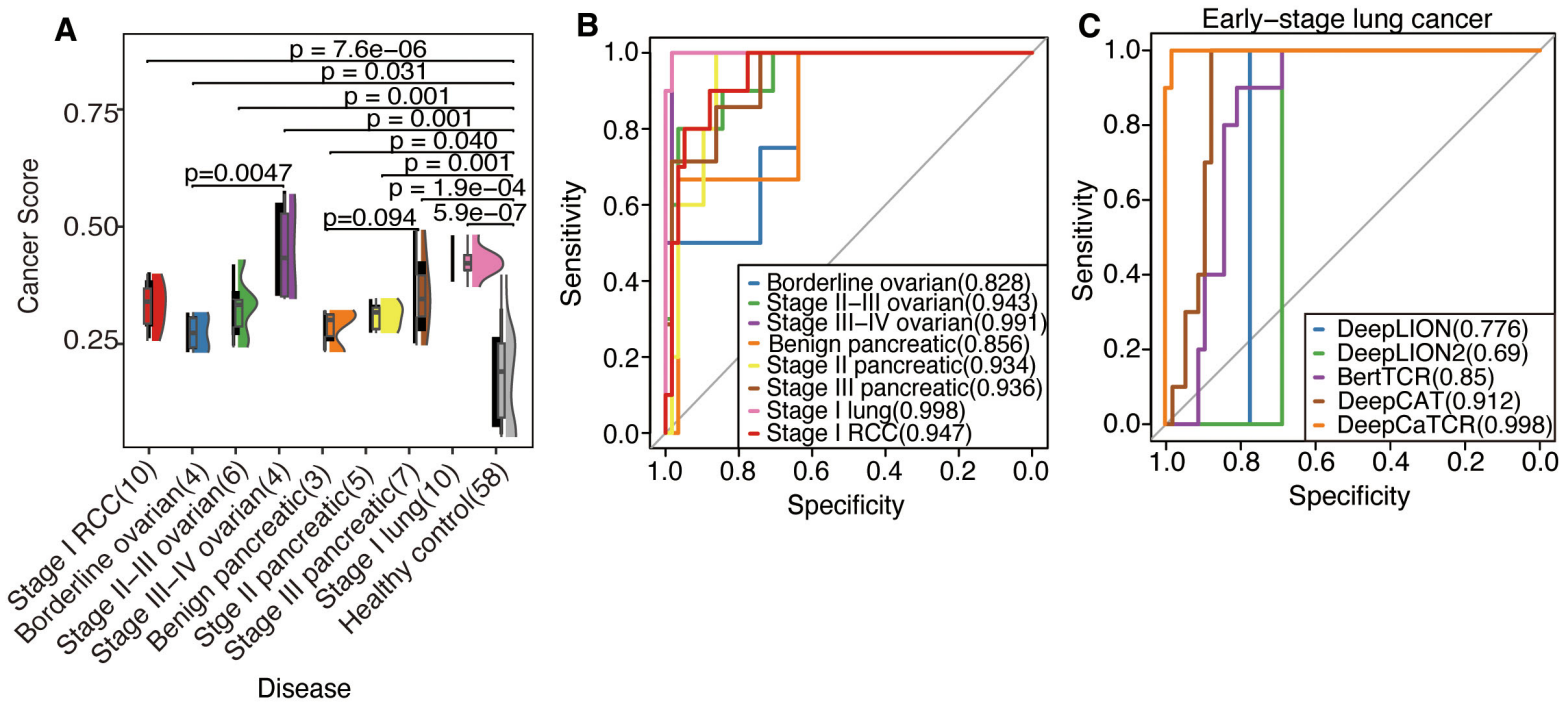
Evaluation of DeepCaTCR’s performance in detecting different stages of cancer. **(A)** The raincloud plot presents cancer scores for various cancer types at different stages in comparison to healthy controls (n = 58). Each cancer group is annotated with its type and stage, along with the sample size in parentheses. p-Values derived from Wilcoxon rank-sum tests, which compare each cancer group to healthy controls, are displayed above each comparison. Kendall’s tau correlation coefficient was employed to evaluate the potential upward or downward trend in cancer score with increasing cancer stage. **(B)** ROC curves for DeepCaTCR across diverse cancer types and stages using the mean scorer. The legend specifies the cancer type and stage, along with the AUC value for each curve. **(C)** ROC curves for different models in early-stage lung cancer. The legend lists the model names and their associated AUC values. ROC, receiver operating characteristic; AUC, area under the receiver operating characteristic curve.

As illustrated in **Figure 4A**, the median cancer score for all early-stage cancers was significantly elevated compared to that of the healthy control group, as determined by the Wilcoxon test (p < 0.05, applicable across all cancer types). Furthermore, Kendall’s tau coefficient demonstrated a positive correlation between cancer scores and disease progression in both ovarian cancer (τ = 0.629, p = 0.0047) and pancreatic cancer (τ = 0.359, p = 0.094). DeepCaTCR achieved high AUCs for different stage cancers (stage I lung: 0.998; stage I RCC: 0.947; stage II pancreatic: 0.934, **Figure 4B**, **Supplementary Table S5**), with specificity consistently >86% across types.

In the context of early-stage lung cancer identification, DeepCaTCR demonstrated superior performance relative to all evaluated benchmarks, as detailed in **Table 3**. DeepCaTCR achieved an AUC of 0.998 (**Figure 4C**), surpassing DeepCAT’s AUC of 0.912, while maintaining a balanced sensitivity of 100% and specificity of 98.3%. In contrast, DeepLION2 and BertTCR exhibited lower specificity at comparable sensitivity levels, with AUCs of 0.69 and 0.85, respectively. The MCC for DeepCaTCR was 0.945, compared to 0.719 for DeepCAT, highlighting its balanced classification performance.

**Table 3:** Performance comparison with different models in early-stage lung cancer.

Model	AUC	ACC	SEN	SPE	F1-score	MCC
DeepLION	0.776	0.809	1.0	0.776	0.606	0.581
DeepLION2	0.69	0.735	1.0	0.69	0.526	0.496
BertTCR	0.85	0.824	0.9	0.8103	0.6	0.5521
DeepCAT	0.912	0.897	1.0	0.879	0.741	0.719
DeepCaTCR	0.998	0.985	1.0	0.983	0.952	0.945

AUC, area under the receiver operating characteristic curve; ACC, accuracy; SEN, sensitivity; SPE, specificity; MCC, Matthews Correlation Coefficient.

### 3.5 Variance-based cancer predictor to enhance early cancer detection performance

**Figure 5:**
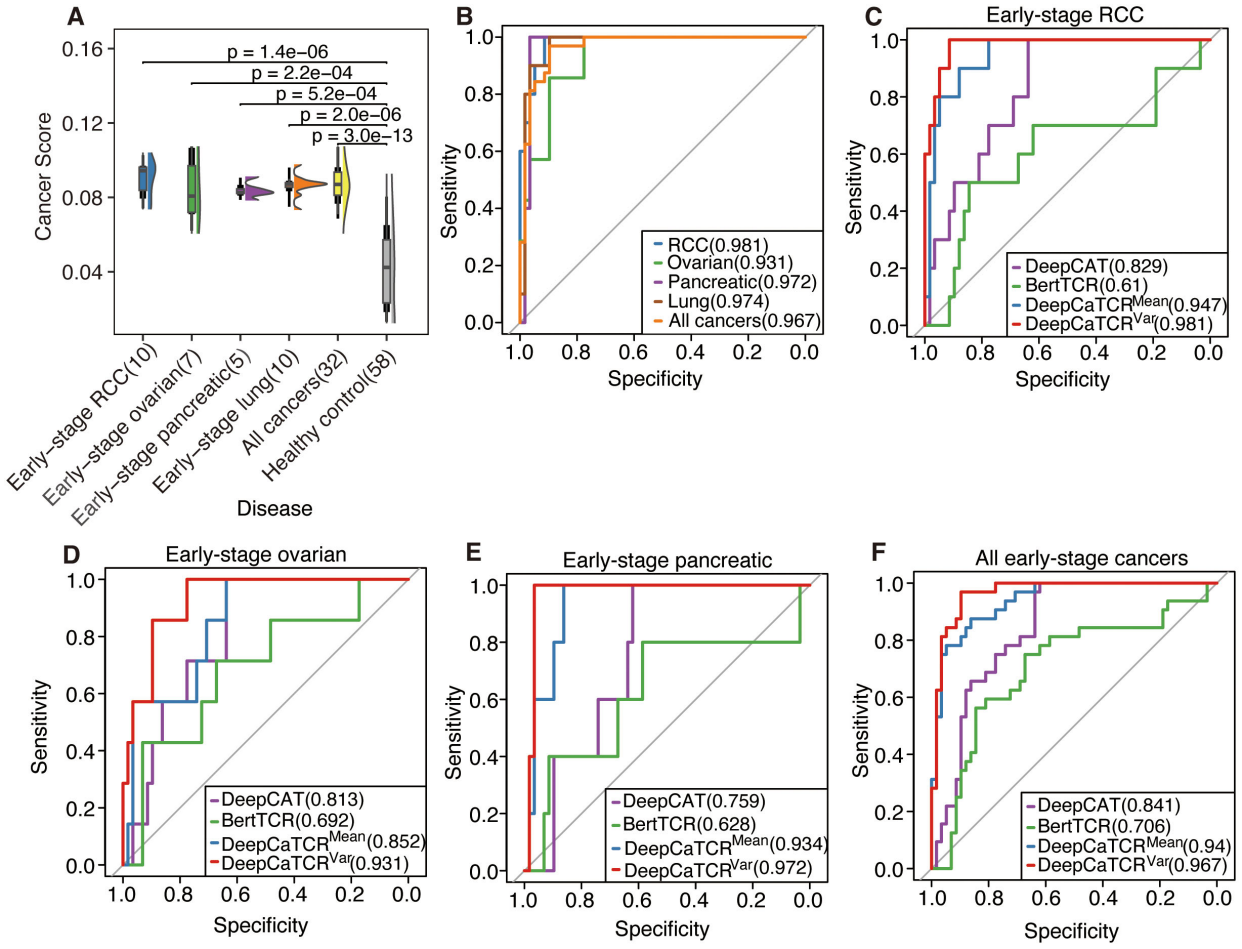
Assessment of DeepCaTCR’s efficacy in identifying early-stage cancer. **(A)** The raincloud plot illustrates cancer scores for early-stage cancer in contrast to healthy controls (n = 58). p-Values obtained from Wilcoxon rank-sum tests comparing each cancer group to healthy controls are indicated above each comparison. **(B)** Evaluation of DeepCaTCR’s capability in early-stage cancer detection using the variance scorer, with ROC curves depicted for various cancer types. (**C–F**) ROC curves for different models in early-stage cancer detection, with the legend providing model names and their corresponding AUC values. ROC, receiver operating characteristic; AUC, area under the receiver operating characteristic curve.

Addressing the limitations inherent in the average scoring strategy for capturing the dynamic characteristics of the TCR repertoire, this study introduces a variance-based repertoire scoring method that markedly enhances the detection performance for early-stage cancers. While average scoring can indicate the overall tumor relevance of the TCR repertoire, it struggles to effectively characterize the heterogeneous features of TCR cancer score distribution during clonal amplification. Consequently, the variance scoring system developed in this study successfully captures the dynamic features of TCR clonal amplification during the early immune response by quantifying the degree of dispersion in cancer score distribution. As illustrated in **Figure 5A**, the distribution of cancer scores among early-stage patients with RCC, OV, PC, and lung cancer exhibited a more pronounced trend of intergroup segregation following the implementation of the variance scoring strategy (p < 0.001). This development facilitated the identification of more discriminative features for the subsequent classification model.

The variance-based DeepCaTCR model demonstrates consistent enhancements in the AUC relative to baseline methodologies (**Table 4**, **Figures 5B–F**). In the context of identifying pancreatic cancer patients, the AUC increased from 0.935 (mean scorer) to 0.972, representing an improvement of ΔAUC = +0.037, while specificity rose from 0.862 to 0.966. For ovarian cancer patient identification, the AUC increased from 0.852 to 0.931, maintaining high specificity (0.776 compared to 0.638 for the average classifier). In the multi-cancer identification task, the unified model achieved an AUC of 0.967, effectively balancing sensitivity (0.969) and specificity (0.897), thereby underscoring its applicability across various cancer types. These findings suggest that variance-based scoring can reduce the false-positive rate, as evidenced by the increased specificity for RCC (0.914 compared to 0.879 with the average scoring model) while preserving sensitivity, which is crucial for early detection.

**Table 4:** Performance comparison with different models in early-stage cancer detection across multiple cancer types.

Disease	Model	AUC	ACC	SEN	SPE	F1-score	MCC
RCC	DeepCAT	0.829	0.691	1.0	0.638	0.488	0.454
	BertTCR	0.61	0.794	0.5	0.845	0.417	0.302
	DeepCaTCR^Mean^	0.947	0.882	0.9	0.879	0.692	0.651
	DeepCaTCR^Variance^	0.981	0.927	1.0	0.914	0.8	0.781
OV	DeepCAT	0.813	0.677	1.0	0.638	0.4	0.399
	BertTCR	0.692	0.677	0.714	0.672	0.323	0.248
	DeepCaTCR^Mean^	0.852	0.677	1.0	0.638	0.4	0.4
	DeepCaTCR^Variance^	0.931	0.8	1.0	0.776	0.519	0.521
PC	DeepCAT	0.759	0.651	1.0	0.621	0.313	0.339
	BertTCR	0.628	0.603	0.8	0.586	0.242	0.21
	DeepCaTCR^Mean^	0.935	0.873	1.0	0.862	0.556	0.576
	DeepCaTCR^Variance^	0.972	0.968	1.0	0.966	0.833	0.831
All cancers	DeepCAT	0.841	0.756	1.0	0.621	0.744	0.607
	BertTCR	0.706	0.7	0.75	0.672	0.64	0.405
	DeepCaTCR^Mean^	0.94	0.867	0.875	0.862	0.824	0.72
	DeepCaTCR^Variance^	0.967	0.922	0.969	0.897	0.899	0.842

AUC, area under the receiver operating characteristic curve; ACC, accuracy; SEN, sensitivity; SPE, specificity; MCC, Matthews Correlation Coefficient; RCC, renal cell carcinoma; OV, ovarian cancer; PC, pancreatic cancer.

At clinically actionable specificity thresholds, variance scores demonstrated exceptional performance (**Table 5**). Specifically, at a specificity level exceeding 98%, variance scores achieved a sensitivity of 62.5%, compared to 53.1% for mean scores, thereby significantly surpassing the performance of DeepCAT (9.4%) and BertTCR (0%). When the specificity threshold was set above 95%, the sensitivity of variance scores increased to 81.3%, in contrast to 75% for the mean score method, indicating their reliability in low-prevalence screening scenarios. Furthermore, the variance-based scoring method exhibited greater robustness in terms of AUC stability, as evidenced by a narrower 95% confidence interval (0.934–0.999) compared to that of the mean-based scoring method (0.895–0.986). The efficacy of the variance-based scoring method may be attributed to its capacity to quantify TCR clonal diversity during the early stages of tumor development, a characteristic that is not captured by mean-based methods.

**Table 5:** Model performance comparison for early-stage cancer detection across different specificity thresholds.

Model	AUC (95% CI)	Sensitivity (specificity > 98%)	Sensitivity (specificity > 95%)	Sensitivity (specificity > 90%)
DeepCAT	0.841 (0.760–0.921)	0.094	0.156	0.312
BertTCR	0.706 (0.587–0.825)	0	0	0.25
DeepCaTCR^Mean^	0.94 (0.895–0.986)	0.531	0.75	0.781
DeepCaTCR^Variance^	0.967 (0.934–0.999)	0.625	0.813	0.875

AUC, area under the receiver operating characteristic curve.

### 3.6 Biological insights into TCR sequences predicted by DeepCaTCR

**Figure 6:**
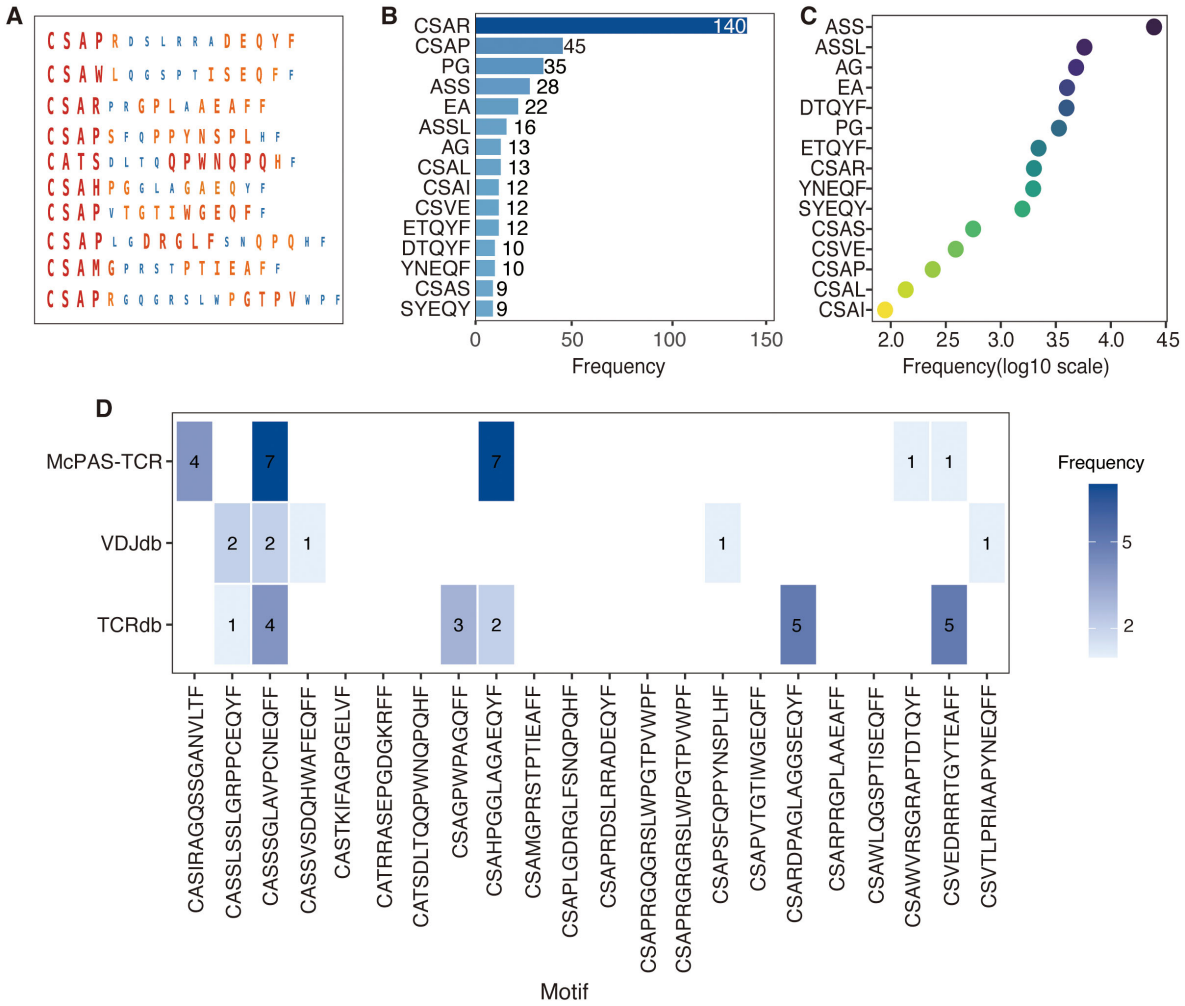
Visualization of motifs and biological insights in DeepCaTCR predicted TCR sequences. **(A)** The visualization of key motifs and their corresponding importance scores within TCR sequences derived from the test set. For each of the 10 representative TCR sequences, the amino acid residues identified as critical motifs by DeepCaTCR are depicted. The size and color intensity of each residue are indicative of its importance score, with larger and darker residues signifying higher scores. **(B)** This panel presents the frequency of key motifs in TCRs with high prediction confidence (prediction score > 0.95). The bar plot provides a summary of the occurrence of top-scoring motifs across TCR sequences with high prediction confidence. **(C)** Frequency of DeepCaTCR-identified key motifs in McPAS-TCR database. A heatmap shows the prevalence of predicted motifs in McPAS-TCR, with darker colors indicating higher occurrence frequencies. **(D)** The overlap between the top 22 high-scoring TCR sequences and known cancer-associated motifs sourced from public databases (TCRdb, VDJdb, and McPAS-TCR). Blue bars represent the number of TCRs that match known cancer-associated motifs, while white bars indicate novel TCRs that do not have matches in the databases. TCR, T-cell receptor.

To elucidate the biological relevance of TCR sequences predicted by DeepCaTCR, key motifs, their functional significance, and overlap with known cancer-associated TCRs were analyzed (**Figure 6**). DeepCaTCR identified key amino acid motifs in TCR sequences from the validation set and assigned importance scores to each motif (**Figure 6A**). Visualization results show that larger and darker residues correspond to higher importance scores, indicating that these sequence patterns may play a key role in antigen recognition. Among TCRs with high prediction confidence (score > 0.95), certain motifs were highly recurrent (**Figure 6B**). The most frequent motifs included “CSAR” (140 occurrences), “CASP” (45 occurrences), and “PG” (35 occurrences). These motifs may represent conserved structural or functional elements in cancer-associated TCRs. A heatmap analysis revealed that DeepCaTCR-identified motifs are enriched in the McPAS-TCR (45) database (**Figure 6C**). “ASS” (24,730 occurrences) and “ASSL” (5,759 occurrences) were among the most frequent motifs in McPAS-TCR, aligning with their high frequency in DeepCaTCR predictions. Other motifs like “AG” (4,831 occurrences) and “EA” (3,997 occurrences) further validated the biological relevance of DeepCaTCR’s predictions.

We initially conducted a search for the top 22 high-scoring TCRs (score > 0.98) across three major databases [TCRdb (46), VDJdb (47), and McPAS-TCR (45)] but did not identify any exact matches among cancer-associated TCRs. This outcome is likely attributable to the exceptionally high diversity of TCR sequences. Considering the significant heterogeneity across cancer types, the absence of these 22 TCRs in existing databases is biologically plausible. To evaluate potential partial matches, we applied various mismatch tolerance criteria tailored to each database’s functionalities. In TCRdb, we recorded near-matches with up to two amino acid mismatches, as provided by the database. VDJdb allowed for the extraction of similar sequences with an Informativeness score of 8 or higher, indicating high-confidence hits. For McPAS-TCR, we conducted local searches using Python scripts, identifying sequences with up to four mismatches, although no hits were found with two or fewer mismatches.

A detailed breakdown is provided in **Figure 6D**, where blue blocks denote partial database matches, such as “CSVEDRRRTGYTEAFF” with five matches in TCRdb and one in McPAS-TCR. The analysis revealed that 11 TCRs (50%) exhibited partial matches in at least one database. For instance, the TCR sequence “CASSSGLAVPCNEQFF” demonstrated four matches in TCRdb and two high-confidence matches in VDJdb, in addition to seven hits in McPAS-TCR. Another TCR, “CSAHPGGLAGAEQYF”, was found to have two matches in TCRdb and seven in McPAS-TCR. Conversely, 11 TCRs (50%) did not exhibit matches in any of the databases under the specified criteria, exemplified by sequences such as “CSAPRDSLRRADEQYF” and “CSARPRGPLAAEAFF”, suggesting that these may represent previously uncharacterized cancer-reactive TCRs.

## 4 Conclusion

In this study, the DeepCaTCR deep learning framework was developed to enhance the recognition specificity of cancer-associated TCRs. This was achieved by integrating a one-dimensional variable convolutional kernel, bidirectional long- and short-term memory units, and a self-attention mechanism, resulting in a discriminative efficacy with an AUC of 0.863 in cross-cancer validation. Additionally, the proposed variance scoring strategy, which is based on TCRβ CDR3 clonal amplification, improved the sensitivity of early-stage cancer detection in peripheral blood to 62.5% by quantifying the heterogeneous features of the immunohistochemical repertoire. This approach achieved an AUC of 0.967 in pan-cancer screening, offering a novel solution to the technical challenge of detecting weak tumor signals in liquid biopsy.

## 5 Discussion

In this study, we developed DeepCaTCR, a deep learning-based framework for TCR repertoire analysis, aimed at improving the efficacy of early cancer detection. A key innovation of this framework is the introduction of a variance-based repertoire scoring strategy, which addresses the limitations of traditional average scoring methods in capturing the dynamic characteristics of immune responses. This novel approach not only enhances the characterization of these dynamics but also establishes a new technical paradigm for pan-cancer early screening. The superior performance of the variance scoring strategy is attributed to its precise modeling of TCR clonal amplification biology. During the initial stages of tumorigenesis, nascent antigen-specific T cells undergo clonal expansion, leading to a highly heterogeneous TCR distribution profile. Our findings indicate that this dynamic evolutionary process is reflected in a significantly greater dispersion in cancer score distribution. In contrast, the conventional mean-value method, by smoothing the data, diminishes the detection sensitivity of this critical biological signal. Through rigorous mathematical modeling and clinical validation, we established a quantitative association between the variance of the TCR distribution and the strength of the tumor immune response.

Despite the advancements achieved, several limitations persist in this study. First, the existing validation predominantly addresses solid tumors, and its applicability to hematological malignancies remains unverified. Second, the occurrence of false positives observed in the HCMV-infected cohort underscores the necessity for an improved background filtering system tailored to infected backgrounds. Lastly, this study utilized retrospective data, necessitating prospective cohort studies to substantiate clinical efficacy. Future research will concentrate on 1) integrating epitope prediction data to refine the variance scoring algorithm, 2) developing a dynamic scoring model informed by longitudinal surveillance, and 3) creating a clinical decision support system to accompany these advancements.

## Data Availability

The original contributions presented in the study are included in the article/**Supplementary Material**. Further inquiries can be directed to the corresponding author.
