# Supplementary material for: Peripheral blood TCR repertoire improves early detection across multiple cancer types utilizing a cancer predictor
Source: Front Oncol. 2025 Aug 27;15:1625369. doi: 10.3389/fonc.2025.1625369 (PMC12420280; doi:10.3389/fonc.2025.1625369)
Supplement: Supplementary file 1 [file DataSheet1.docx]

Supplementary Material

The PDF file includes:

**Figure S1.** The performance metrics derived from a 5-fold cross-validation analysis.

**Figure S2.** A correlation analysis between cancer risk scores and TCR counts in healthy individuals.

**Table S1.** The details of the dataset used in this study.

**Table S2.** The performances of DeepCaTCR across hyperparameter tuning configurations.

**Table S3.** Performance of DeepCaTCR in identifying cancer patients across sample sources and disease types.

**Table S4**. Performance of DeepCaTCR in distinguishing cancer patients from individuals with different viral infections or healthy cohorts.

**Table S5.** Diagnostic performance of DeepCaTCR across cancer types and stages.


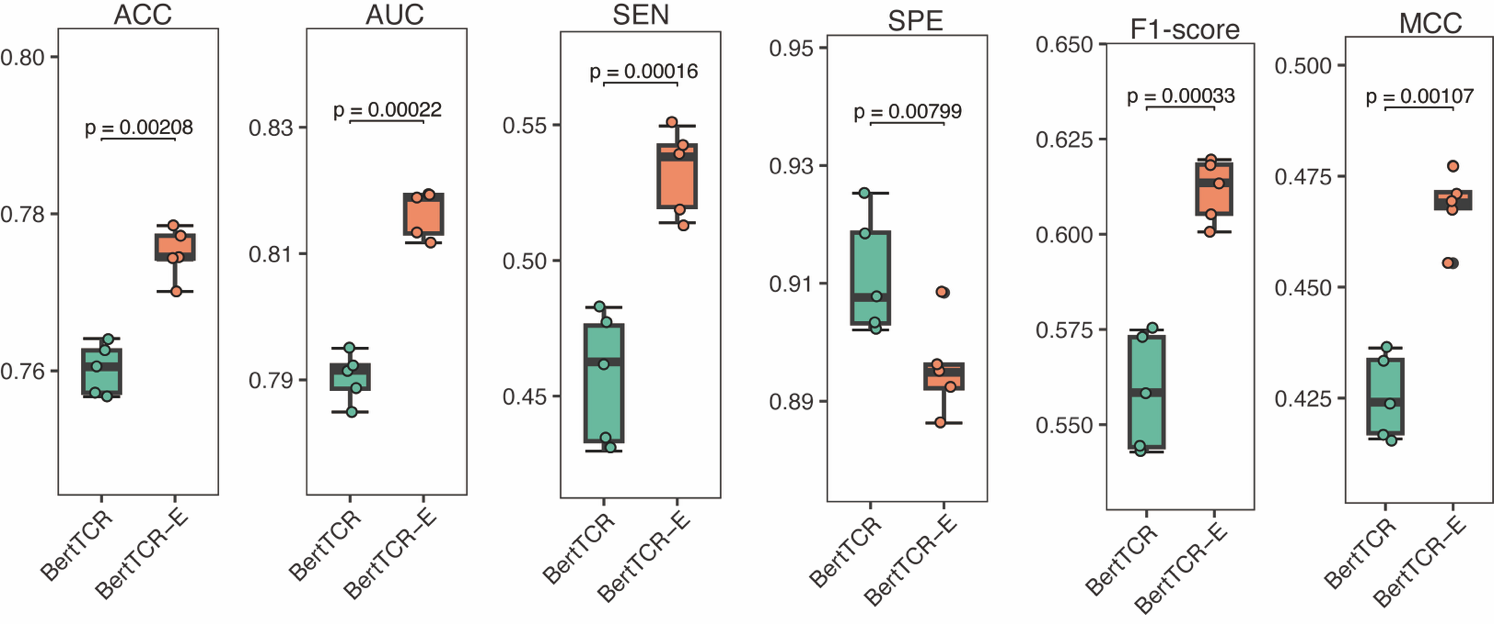


**Supplementary Figure S1.** The performance metrics derived from a 5-fold cross-validation analysis. The p-values were obtained through paired t-tests comparing the performance metrics of BertTCR and BertTCR-E.


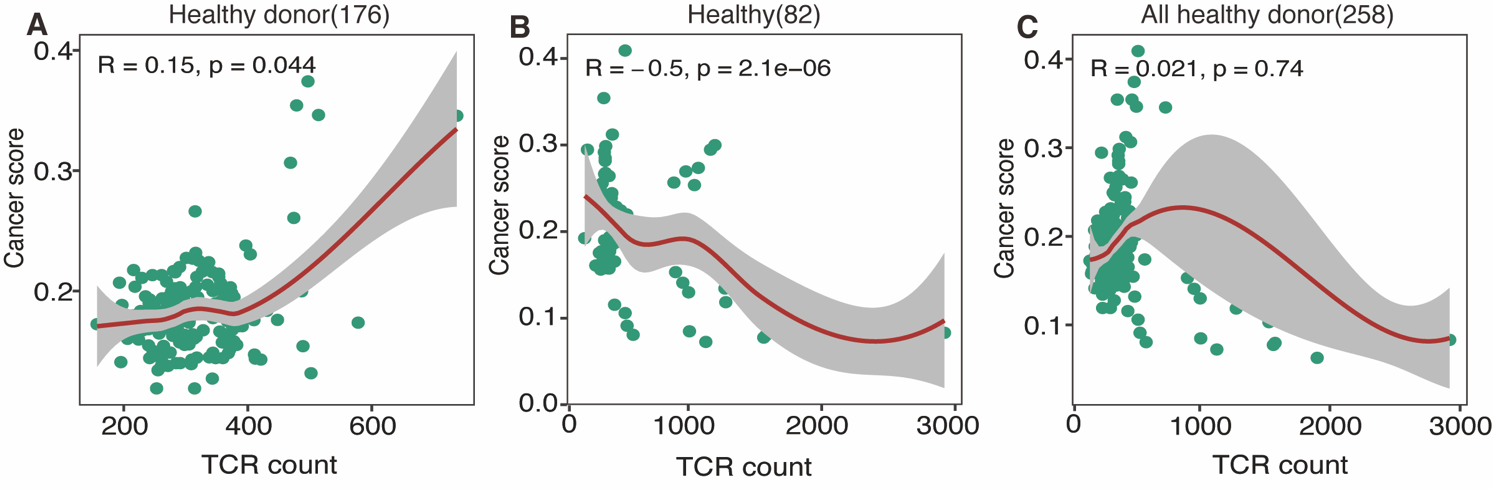


**Supplementary Figure 2.** A correlation analysis between cancer risk scores and TCR counts in healthy individuals. (**A**) A scatter plot illustrating the relationship between TCR counts and cancer risk scores in a cohort of healthy participants (n = 176), accompanied by a Lowes smooth curve and a 95% confidence interval. The Spearman correlation coefficient (R) and its statistical significance are provided in the inset. (**B**) A comparable scatter plot for a separate cohort of healthy participants (n = 82). (**C**) The scatter plot for the aggregated cohort of healthy participants (n = 258).

**Supplementary Table S1**. The details of the dataset used in this study.

| Data type | Dataset | Disease | Sample number | Cell type | Usage in this paper |
| --- | --- | --- | --- | --- | --- |
| Train data | TCGA | Multiple cancers | 4200 | TIL | Figure 2 |
|  | IA | Non-cancer | 120 | PBMC |  |
| Test data | IA | Breast | 16 | TIL | Figure 3A-B |
|  |  | Lung BM | 20 | TIL |  |
|  |  | Lung | 20 | TIL |  |
|  |  | Melanoma | 23 | TIL |  |
|  |  | Pancreatic | 9 | TIL |  |
|  |  | Melanoma | 21 | PBMC | Figure 3C-D, Figure 4A-B |
|  |  | Early-stage breast | 16 | PBMC |  |
|  |  | Stage III-IV ovarian | 4 | PBMC |  |
|  |  | Stage III pancreatic | 7 | PBMC |  |
|  |  | CRC | 3 | PBMC | Figure 3E |
|  |  | Bladder | 30 | PBMC |  |
|  |  | GBM | 15 | PBMC |  |
|  |  | Lung | 29 | PBMC |  |
|  |  | YFV | 9 | PBMC | Figure 3F-G |
|  |  | GVHD | 15 | PBMC |  |
|  |  | HCMV | 179 | PBMC |  |
|  |  | Healthy | 24 | PBMC |  |
|  |  | Healthy donor | 176 | PBMC | Figure 3A-I |
|  |  | Stage I-II lung | 11 | PBMC | Figure 4A-B, C, Figure 5A-B, F |
|  |  | Stage III lung | 4 | PBMC | Figure 4A-B, |
|  |  | Healthy control | 58 | PBMC | Figure 4A-B, Figure 5A-F |
|  | Beshnova et al., 2020 | Stage I RCC | 10 | PBMC | Figure 4A-B, Figure 5A-C |
|  |  | Borderline ovarian | 4 | PBMC | Figure 4A-B, Figure 5A-B, D |
|  |  | Stage II-III ovarian | 6 | PBMC | Figure 4A-B |
|  |  | Stage II pancreatic | 5 | PBMC | Figure 4A-B, Figure 5A-B, E |

TCGA, The Cancer Genome Atlas; IA, Adaptive Biotechnologies immuneACCESS online database; TIL, tumor-infiltrating T lymphocyte; PBMC, peripheral blood mononuclear cell; Lung BM, Lung cancer and brain metastasis; CRC, Colorectal cancer; GBM, Glioblastoma Multiforme; YFV, Yellow Fever Virus; GVHD, Graft-versus-Host Disease; HCMV, Human Cytomegalovirus; RCC, Renal Cell Carcinoma.

**Supplementary Table S2**. The performances of DeepCaTCR across hyperparameter tuning configurations.

| Learning rate | Dropout | Head | k-max pooling | Kernel size | Kernel number | ACC | AUC | SEN | SPE | F1 | MCC |
| --- | --- | --- | --- | --- | --- | --- | --- | --- | --- | --- | --- |
| 0.001 | 0.3 | 2 | 3 | 2,3,4,5 | 4,4,4,4 | 0.800 ± 0.009 | 0.854 ± 0.012 | 0.621 ± 0.012 | 0.890 ± 0.012 | 0.672 ± 0.016 | 0.534 ± 0.025 |
| 0.01 | 0.3 | 2 | 3 | 2,3,4,5 | 4,4,4,4 | 0.718 ± 0.027 | 0.706 ± 0.068 | 0.349 ± 0.111 | 0.902 ± 0.021 | 0.443 ± 0.100 | 0.302 ± 0.080 |
| 0.001 | 0.4 | 2 | 3 | 2,3,4,5 | 4,4,4,4 | 0.784 ± 0.044 | 0.824 ± 0.079 | 0.523 ± 0.179 | 0.915 ± 0.020 | 0.600 ± 0.160 | 0.482 ± 0.140 |
| 0.01 | 0.4 | 2 | 3 | 2,3,4,5 | 4,4,4,4 | 0.732 ± 0.010 | 0.737 ± 0.018 | 0.408 ± 0.010 | 0.897 ± 0.015 | 0.505 ± 0.005 | 0.354 ± 0.018 |
| 0.001 | 0.5 | 2 | 3 | 2,3,4,5 | 4,4,4,4 | 0.807 ± 0.003 | 0.864 ± 0.003 | 0.586 ± 0.026 | 0.918 ± 0.009 | 0.669 ± 0.012 | 0.548 ± 0.009 |
| 0.01 | 0.5 | 2 | 3 | 2,3,4,5 | 4,4,4,4 | 0.730 ± 0.023 | 0.741 ± 0.034 | 0.348 ± 0.114 | 0.903 ± 0.042 | 0.458 ± 0.103 | 0.355 ± 0.059 |
| 0.001 | 0.5 | 2 | 3 | 2,3,4 | 4,4,4 | 0.736 ± 0.132 | 0.777 ± 0.155 | 0.551 ± 0.031 | 0.827 ± 0.183 | 0.617 ± 0.066 | 0.413 ± 0.231 |
| 0.001 | 0.5 | 2 | 3 | 2,3,4,5,6 | 4,4,4,4,4 | 0.806 ± 0.005 | 0.847 ± 0.002 | 0.550 ± 0.029 | 0.906 ± 0.010 | 0.641 ± 0.006 | 0.521 ± 0.006 |
| 0.001 | 0.5 | 2 | 2 | 2,3,4,5 | 4,4,4,4 | 0.795 ± 0.010 | 0.846 ± 0.014 | 0.586 ± 0.023 | 0.899 ± 0.014 | 0.655 ± 0.016 | 0.520 ± 0.022 |
| 0.001 | 0.5 | 2 | 4 | 2,3,4,5 | 4,4,4,4 | 0.791 ± 0.007 | 0.836 ± 0.006 | 0.585 ± 0.017 | 0.883 ± 0.018 | 0.647 ± 0.009 | 0.507 ± 0.011 |
| 0.001 | 0.5 | 2 | 5 | 2,3,4,5 | 4,4,4,4 | 0.727 ± 0.012 | 0.741 ± 0.011 | 0.582 ± 0.020 | 0.892 ± 0.018 | 0.547 ± 0.019 | 0.507 ± 0.026 |
| 0.001 | 0.5 | 1 | 3 | 2,3,4,5 | 3,3,3,3 | 0.783 ± 0.006 | 0.832 ± 0.012 | 0.604 ± 0.022 | 0.884 ± 0.009 | 0.642 ± 0.009 | 0.497 ± 0.017 |
| 0.001 | 0.5 | 1 | 3 | 2,3,4,5 | 5,5,5,5 | 0.764 ± 0.009 | 0.803 ± 0.011 | 0.523 ± 0.046 | 0.882 ± 0.008 | 0.587 ± 0.017 | 0.450 ± 0.025 |
| 0.001 | 0.5 | 1 | 3 | 2,3,4,5 | 2,2,2,2 | 0.790 ± 0.006 | 0.842 ± 0.006 | 0.598 ± 0.020 | 0.886 ± 0.007 | 0.655 ± 0.012 | 0.511 ± 0.013 |
| 0.001 | 0.5 | 1 | 3 | 2,3,4,5 | 4,4,4,4 | 0.780 ± 0.006 | 0.827 ± 0.010 | 0.585 ± 0.023 | 0.877 ± 0.015 | 0.638 ± 0.011 | 0.486 ± 0.014 |
| 0.001 | 0.5 | 0 | 1 | 2,3,4 | 3,3,3 | 0.775 ± 0.003 | 0.817 ± 0.004 | 0.533 ± 0.015 | 0.896 ± 0.008 | 0.611 ± 0.008 | 0.468 ± 0.008 |

**Supplementary Table S3**. Performance of DeepCaTCR in identifying cancer patients across sample sources and disease types.

| Sample source | Disease | AUC | ACC | SEN | SPE | F1-score | MCC |
| --- | --- | --- | --- | --- | --- | --- | --- |
| TIL | Breast cancer | 0.983 | 0.948 | 1.0 | 0.943 | 0.762 | 0.762 |
|  | Lung BM | 0.95 | 0.995 | 0.95 | 1.0 | 0.974 | 0.972 |
|  | Lung cancer | 1.0 | 1.0 | 1.0 | 1.0 | 1.0 | 1.0 |
|  | Melanoma | 0.994 | 0.965 | 1.0 | 0.96 | 0.868 | 0.858 |
|  | Pancreatic | 0.998 | 0.989 | 1.0 | 0.989 | 0.9 | 0.899 |
| PBMC | Breast cancer | 0.955 | 0.912 | 0.938 | 0.909 | 0.638 | 0.636 |
|  | Melanoma | 0.961 | 0.924 | 0.952 | 0.921 | 0.727 | 0.713 |
|  | Ovarian | 0.997 | 0.994 | 1.0 | 0.994 | 0.889 | 0.892 |
|  | Pancreatic | 0.989 | 0.962 | 1.0 | 0.96 | 0.667 | 0.693 |
|  | CRC | 0.919 | 0.799 | 1.0 | 0.796 | 0.143 | 0.247 |
|  | Bladder | 0.83 | 0.82 | 0.8 | 0.824 | 0.565 | 0.497 |
|  | GBM | 0.814 | 0.565 | 1.0 | 0.528 | 0.266 | 0.284 |
|  | Lung cancer | 0.667 | 0.722 | 0.552 | 0.75 | 0.36 | 0.231 |

**Supplementary Table S4**. Performance of DeepCaTCR in distinguishing cancer patients from individuals with different viral infections or healthy cohorts.

| Disease | AUC | ACC | SEN | SPE | F1-score | MCC |
| --- | --- | --- | --- | --- | --- | --- |
| YFV | 0.992 | 0.986 | 0.985 | 1.0 | 0.993 | 0.898 |
| GVHD | 0.984 | 0.98 | 0.985 | 0.933 | 0.989 | 0.893 |
| HCMV | 0.956 | 0.905 | 0.912 | 0.899 | 0.893 | 0.808 |
| Healthy donor | 0.978 | 0.949 | 0.942 | 0.955 | 0.942 | 0.896 |
| Additional healthy samples | 0.947 | 0.868 | 0.854 | 0.89 | 0.89 | 0.729 |

**Supplementary Table S5**. Diagnostic performance of DeepCaTCR across cancer types and stages.

| Disease | AUC | ACC | SEN | SPE | F1-score | MCC |
| --- | --- | --- | --- | --- | --- | --- |
| Borderline ovarian | 0.828 | 0.661 | 1.0 | 0.638 | 0.276 | 0.32 |
| Stage II-III ovarian | 0.943 | 0.941 | 0.8 | 0.966 | 0.8 | 0.766 |
| Stage III-IV ovarian | 0.991 | 0.984 | 1.0 | 0.983 | 0.889 | 0.887 |
| Benign pancreatic | 0.856 | 0.656 | 1.0 | 0.638 | 0.222 | 0.282 |
| Stage II pancreatic | 0.935 | 0.873 | 1.0 | 0.862 | 0.556 | 0.576 |
| Stage III pancreatic | 0.936 | 0.769 | 1.0 | 0.741 | 0.483 | 0.486 |
| Stage I lung | 0.998 | 0.985 | 1.0 | 0.983 | 0.952 | 0.945 |
| Stage I RCC | 0.947 | 0.882 | 0.9 | 0.879 | 0.692 | 0.651 |
